# Genetic Therapy of Fuchs Endothelial Corneal Dystrophy: Where Are We? A Review

**DOI:** 10.3390/genes16101222

**Published:** 2025-10-15

**Authors:** Spela Stunf Pukl

**Affiliations:** 1 Medical Faculty, University of Ljubljana, 1000 Ljubljana, Slovenia; spela.stunf@siol.net; Tel.: +386-41-382-487; 2 Eye Hospital, University Clinical Center Ljubljana, 1000 Ljubljana, Slovenia

**Keywords:** Fuchs endothelial corneal dystrophy (FECD), genetic therapy, CRISPR-Cas9

## Abstract

Objectives: The incidence of Fuchs endothelial corneal dystrophy (FECD) is growing, and with it, the unmet need for a corneal transplant. Among alternative treatment modalities, only genetic therapy represents a causal therapy. Methods: Following the SNARA protocol, the PubMed and ClinicalTrials databases were searched using the keywords Fuchs endothelial corneal dystrophy, FECD, genetic therapy, and CRISPR-Cas9. Results: FECD is polyfactorial disease and mutations or polymorphisms in at least 15 different genes were connected to the disease. For the early-onset form of the disease, exclusive connection to mutations in *COL8A2* was confirmed, while for the late-onset form, the most characteristic mutation is the expansion of the CTG18.1 triplet in the *TCF4* gene, making these two possible targets. While the CRISPR-Cas9 approach represents the mainstay of genetic therapy development recently, the application of this method to FECD contains several obstacles, studied in preclinical settings. DT-168 and the Ad-Cas9-Col8a2gRNA molecules were developed for FECD treatment and preclinically tested, and phase I and II clinical studies for DT-168 are also already being performed. Conclusions: The review of the literature proved that genetic therapy for FECD is at the level of preclinical research and that there are several specific challenges connected to the target genetic mutation as well as the delivery of possible treatment and duration of the effect. Further studies in the field might bring solutions in the future for alternative treatments for this common corneal disease.

## 1. Introduction

**Figure 1:**
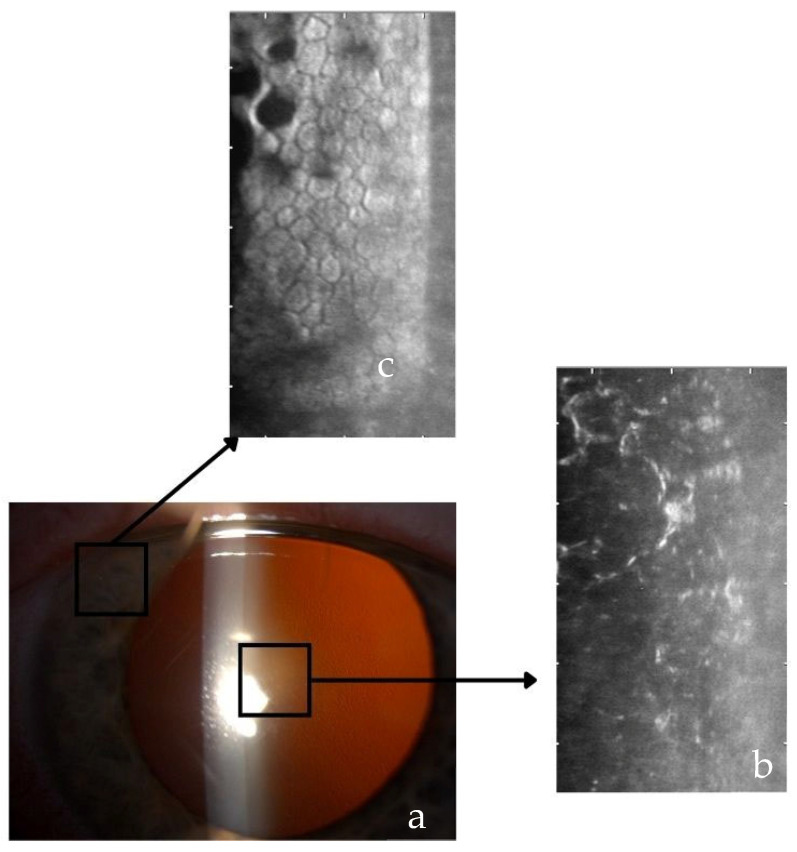
Photograph of cornea in a patient with Fuchs endothelial corneal dystrophy; grade 3; endothelial corneal changes are present in the central cornea (**a**). Specular microscopy of corneal endothelium in the (**b**) center showing the image of changed and dysfunctional endothelial cells, and the (**c**) periphery with normal endothelium. The patient with normal periphery can be treated with Descemet stripping only (DSO) procedure. Source: unpublished collection from archive of Eye Hospital University Clinical Center Ljubljana.

The Fuchs endothelial corneal dystrophy (FECD, *MIM 136800*) was introduced by Ernst Fuchs in 1910. It is a frequent progressive late-onset genetic and multifactorial disease, affecting primary corneal endothelium. Patients with FECD experience debilitating vision loss due to progressive corneal opacification resulting from endothelial cell loss. Endothelial changes commence in the central cornea, and progress towards the periphery (Figure 1). Dysfunctional endothelium eventually causes corneal edema [1].

FECD represents the number one cause for corneal transplantation in the developed world; endothelial transplantation represents the golden standard of care for patients with FECD. With this procedure, healthy donor endothelium is transplanted selectively in order to regain the endothelial pump function and cornel transparency. An aging population causes growing numbers of FECD, and cornea clinics are challenged due to a constant shortage of corneal tissue and consequent long waiting times for transplantation, which results in the progression of the disease to persistent corneal edema and secondary stromal fibrosis, which is the stage where endothelial transplantation is not sufficient for visual recovery.

This is the major reason which drives several different initiatives for alternatives to transplantation. Clinically, pharmacological endothelial corneal regenerative therapy with Rho kinase inhibitors is being studied in this respect [1] and there were a series of patients already treated with cell therapy [2,3].

Mirroring the other ophthalmologic genetic treatments already approved for clinical use [4], FECD is being studied for genetic treatment. This review article aims to present the current status and future pipeline of genetic therapy for FECD.

## 2. Methods

To review the current knowledge in the field of genetic therapy for FECD, the Scale for the Assessment of Narrative Review Articles—SNARA protocol was used.

The PubMed and ClinicalTrials databases were searched for preclinical as well as clinical reports.

The keywords used for searching the publications were as follows: Fuchs endothelial corneal dystrophy, FECD, genetic therapy, CRISPR-Cas9 in the period 2000 to 2025.

## 3. Fuchs Endothelial Corneal Dystrophy

### 3.1. Clinical Staging and Symptomatic

The main clinical findings in early FECD are guttae on the endothelial side of the cornea surface, which are composed of excessive extracellular matrix. The Descemet membrane is changed and thickened, and there is secondary endothelial cell layer disruption [5]. The main symptom of early stage FECD is blurring of vision; some patients also report glare, and about one third are asymptomatic [6,7].

Despite the absence of epithelial or stromal edema, the light scattering by guttae can cause debilitating symptoms, influence the quality of life, and warrants treatment at an early stage of the disease [6,8].

Later stages of the disease are characterized by corneal edema due to severe endothelial function loss [5].

Firstly, the symptoms are worse in the morning, and later the vision is continuously blurred [6].

The typical presentation of FECD is the late-onset subtype of the disease, which clinically presents between the fifth and seventh decade of life. The early-onset subtype, which typically occurs in the first decade of life, represents less than 5% of cases [7].

FECD is overall more frequent and progressive in females; however, if only the late-onset form is considered, the male population is affected three times more often [7,9].

### 3.2. Etiology of FECD

The etiology of FECD is complex and heterogenic with genetic, lifestyle, and environmental factors influencing its occurrence and course [10,11,12].

## 4. Genetics of FECD

### 4.1. Inheritance of FECD

The inheritance is autosomal dominant with incomplete penetrance and variable expressivity; thus, there is a vast clinical spectrum of age of onset, symptoms severity, and progression even among individuals within a single family [11].

Corneal clinics are even more challenged due to the fact that about half of the cases occur sporadically, and only half are familial [10].

### 4.2. FECD Genes

There are more than 15 genes with mutations and/or single-nucleotide polymorphisms associated with FECD [10] (Table 1).

The early-onset subtype differs from the late-onset genetically.

#### 4.2.1. Early-Onset Subtype

For the early-onset subtype, the mutations in the *COL8A2* gene are characteristic. Mutations causing early-onset FECD have been exclusively linked to the α2 chain of collagen 8 *COL8A2* [10].

The *COL8A2* gene is the only gene that has been associated with early-onset FECD and is rarely associated with late-onset FECD [13].

A genome-wide study proved different pathogenic variants in *COL8A2* on chromosome 1p34.3-p32: R304Q, R434H, Q455K, and R155Q, [13]; the latter was proved as non-pathogenic later [14].

Mutations in the *COL8A* gene in the early-onset subtype affect the structure of the Descemet’s membrane. The *COL8A2* gene encodes the 703 amino acid α_2_ subunit of collagen VIII, which is a short-chain collagen that is a component of Descemet membrane secreted by CECs [13].

#### 4.2.2. Late-Onset Subtype

On the other hand, the far more frequent form, the late-onset FECD, is most characteristically underlined by the expansion of the CTG18.1 triplet in the *TCF4* gene [10,40,41,42].

Namely, the mutations in the *TCF4* gene are present in 70% of Caucasians with FECD.

Apart from these, *SLC4A11* variants were proved to be another possible genetic cause, while the causal role of other candidates, such as *ZEB1/TCF8*, *LOXHD1*, and *AGBL1*, need still to be confirmed [17,43]. Some less common genetic mutations were also described in *COL8A2*, *DMPK*, *KANK4*, *LAMC1*, *ATP1B1*, *RAD51*, *FEN1*, *XRCC1*, *NEIL1*, *TGFBI*, *CLU*, *PITX2*, *PTPRG*, *FASLG*, and *KCNJ13* [5].

The strongest genetic association with the clinical picture of FECD was proved for the CTG trinucleotide repeat within the transcription factor 4 (*TCF4*); a repeat length >50 was highly specific for the disease [18,42,43,44]. Following this significant correlation, expansion of this CTG repeat might be important in the mainstay of patients with developed disease. In early stages or clinically nonsignificant grades of pathological endothelial changes, diagnosing the trinucleotide expansion could help to predict the disease and lead to early therapeutic approach with minimally invasive techniques [18,44].

The genetic locus of early-onset and late-onset FECD have been mapped to different chromosomes (Table 2) [45].

##### *TCF4* Gene

Most cases of FECD (late-onset) are caused by the expansion of trinucleotide repeats in the *TCF4* gene. The expansion of greater than 40–50 repeats results in the production and accumulation of RNA inside the endothelial cell nuclei in the so-called RNA foci [21,46]. The toxic RNA causes sequestration of muscle blind-like protein MBNL-1 and MBNL2, splicing regulator proteins in nuclear RNA focuses. As a result of its loss of function, several miss-splicing transcripts involved in endothelial corneal cell homeostasis occur, leading to cell dysfunction and deterioration. The severity of the disease correlates to the length of the repeat [47].

As detailed in a comprehensive review of the research implicating CTG18.1 expansion in disease pathogenesis, the proposed mechanisms by which the CTG18.1 expansion may drive or exacerbate the onset of FECD include *TCF4* dysregulation, toxic gain-of-function from *TCF4* repeat-containing RNA, toxic gain-of-function from repeat-associated non-AUG-dependent translation, and somatic instability of CTG18.1 [41].

The CTG18.1 trinucleotide repeat is the most common genetic background in patients with FECD and can be confirmed in as frequent as 70–80% of individuals with pathological corneal findings [41], which is not the case for any other FECD-associated genetic mutations. Furthermore, the mutation has a direct pathogenic mechanism leading to cell deterioration and has already been studied in clinical settings as a possible diagnostic and prognostic biomarker [12]. The listed facts make this mutation a feasible option for the targeting of genetic therapy.

However, there are also several features specific to this mutation that need to be addressed, before a successful therapeutic strategy can be established. The trinucleotide repeat is placed in the intron 3 of TCF4 and thus does not result in an altered protein but rather, results in toxic RNA, for which a classic genetic gene replacement is not an option.

Large, repetitive DNA tracts also pose different problems for CRISPR-Cas9 editing, due to inefficient binding, possible off-target cleavage, difficulty in completing the excision of a large repeats, and unreliable repair mechanisms in non-dividing endothelial cells [41].

##### *SLC4A11* Gene and Other

The *SLC4A11* gene encodes an ion channel that promotes water resorption through the endothelial layer and is an important mediator of solute transport in the cornea.

Mutations in this gene can lead to corneal edema and correlate with FECD.

Similarly, mutations in the *ZEB1* gene have been associated with late-onset of FECD and posterior polymorphous corneal dystrophy [48,49].

## 5. Genetic Treatment for FECD

The cornea seems like a possible target for developing genetic treatments as it is an immune-privileged, avascular, transparent, and relatively accessible tissue.

There are several well-established surgical techniques offering access to and manipulation of cells and layers of the cornea, but an efficient and targeted delivery of genetic material exactly to the corneal endothelial cells represents an additional new challenge. Challenges in establishing possible corneal genetic therapy include but are not limited to achieving sustained non-pathological gene transcription and minimizing off-target effects.

### 5.1. Established Treatments for FECD

To regain endothelial function and corneal transparency in patients with FECD, endothelial cells need to be repopulated in the areas of the diseased Descemet and dysfunctional or absent endothelial cells.

**Figure 2:**
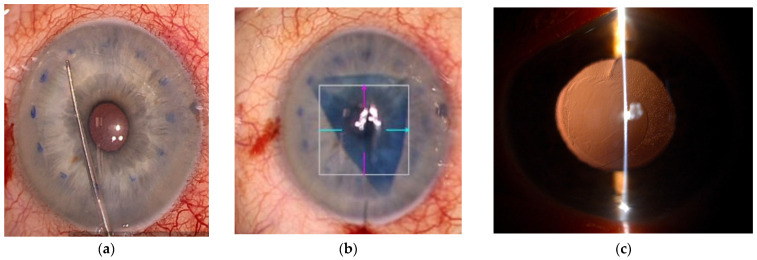
Surgical procedures for FECD—(**a**,**b**) Descemet membrane endothelial keratoplasty (DMEK) intraoperative view: (**a**) 8 mm descemetorhexis on recipient eye, (**b**) endothelial graft was inserted into the anterior chamber, (**c**) central diseased endothelium was removed during Descemetorhexis only (DSO)—retroillumination view of the transparent cornea after healing due to peripheral endothelial cell movement into the central part of the cornea. Source: archive, unpublished, collection from archive of Eye Hospital University Clinical Center Ljubljana.

The golden standard of care is endothelial transplantation (Figure 2a) [50,51,52].

This is successful but technically demanding with surgical risks [53] of possible graft failure or rejection [54,55]. Above all, the constant global tissue shortage [56,57] results in prolonged waiting times for surgery with possible aggravation of the cornea status during the waiting period, resulting in more demanding treatment and worse outcomes [58,59].

Thus, several treatments that do not need a tissue transplantation emerged, including Descemetorhexis only (DSO) (Figure 2c) for candidates with preserved peripheral endothelium (Figure 1) [60,61], pharmacological regeneration [60,61], and cell therapy.

### 5.2. Genetic Therapy Principles in FECD

Since FECD is linked to specific mutations and the cornea has an immuno-privileged and relatively accessible position, it might be a possible disease for genetic therapy once the biological and technical challenges, including ensuring an efficient and targeted delivery of genetic material to the corneal endothelial cells, sustained gene expression, and minimal off-target effects, are successfully addressed.

Genetic treatment, which will only become clinically accepted following long and precise preclinical research steps, should, in general, be focused on the most prevalent genetic background of the disease.

For genetic therapy of FECD, the most possible target seems to be the *TCF4* gene. Namely, the far more prevalent subtype of FECD is the late-onset form and for this subtype, it has been proven to be connected to four genetic loci (*FCD1-4*), different probable loci, and point mutations in a minimum of three other genes (*ZEB1*/*TCF8*, *SLC4A11*, and *LOXHD1*). Of these, the trinucleotide repeat in the *TCF4* gene was shown to be the genetic cause of the majority (70%) of FECD cases [47].

Traditional gene therapy typically involves delivering functional copies of a gene to compensate for the effects of a mutated or non-functional version [62].

New approaches in molecular biology tend to repair the pathological genetic mutation in situ. Nucleases precisely cut targets in the host genome, which is the beginning of genome-editing.

Of them, the regularly clustered interspaced short palindromic repeats/CRISPR-associated protein 9 (CRISPR-Cas9) system, which was originally discovered as the adaptive immune system of bacteria, reigns as a preeminent innovative technology [63] in human biomedicine due to its precision, high adaptability, programmability, efficacy, and relative cost effectiveness.

Namely, the gRNA in the Cas9-gRNA complex guides the Cas9 to cleave the DNA at a specific target location(s). Cas9 induces a double-stranded DNA break, followed by activation of cell mechanisms to repair the DNA by either non-homologous end joining (NHEJ) or homology-directed repair (HDR) pathways [64].

The CRISPR-Cas9 system proved successful in treating inherited retinal disorders [65]. Namely, a normal *CEP290* gene expression was regained in an in vivo mouse study with Leber congenital amaurosis [66], followed by a clinical trial to evaluate the safety and effectiveness of EDIT-101, which is an experimental gene editing treatment [67]; approved gene therapy was later conducted for patients with biallelic mutations in *RPE65* (Luxturna, Hoffmann-La Roche, Basel, Switzerland) [68].

However, use of this technology on the trinucleotide repeat diseases, such as FECD, presents specific and additional challenges.

### 5.3. Challenges of the CRISPR-Cas9 System in General

The main obstacle to clinical use is the efficient and precise delivery of the CRISPR-Cas9 complex to the target cells.

The golden standard of the delivery system in eye genetic therapy is recombinant adeno-associated viruses (AAVs). They are nonintegrating, with lower risk of unwanted mutation in host genome. There is a potential loss of effect over time, especially in dividing cells. An example of eye genetic treatment, that is AAV-based is the genetic therapy for retinitis pigmentosa, which has been approved by the FDA [69].

The capacity of AAV vectors is very limited and represents a limiting factor for the delivery, especially due to large size of the Cas9 complex [70]. For the genetic treatment of retinal diseases, solutions like smaller caspases [71] or dual-AAV split system [72,73] were introduced.

In any clinical use, the precise modification of genes is one of the most important safety concerns; thus, the possible off-target effects of the CRISPR-Cas9 therapy have to be solved in preclinical research [74]. Several technologies of tissue function have already been developed [75,76,77]. There is also existing software to predict possible off-target binding of RNA-guided endonucleases using sgRNA target sequences and the host genome. However, the significant variability of experimentally predicted verified off-target binding sites was proved [78]. Namely, in the field of ocular genetic CRISPR therapy, off-target control is based on guide selection [79], high-fidelity Cas9 variants [75,80] (like SpCas9, Cas9-HF, HypaCas9, and SniperCas9 [81]), dual-nickase or base/prime editing approaches [82], restricted Cas9 expression in tissue [83], and rigorous preclinical off-target profiling [79,84]. Before any clinical use, the off-target editing is further denied with the aid of preclinical safety studies [85].

Eye and ocular structures and tissues have immune-privileged status; however, due to the reported immunoreactivity to Cas9, validation of the safety of CRISPR-Cas9-based therapeutics in the clinical real world must be performed [69].

#### Specific Challenges of Applying CRISPR-Cas9 to TCF4

As discussed in Section 4.2.2, the most common mutation found in FECD is the expansion of trinucleotide repeats in the *TCF4* gene with consequent production and accumulation of toxic RNA inside endothelial cell nuclei, and sequestration of splicing regulator protein, resulting in cell dysfunction and deterioration. The pathogenic trinucleotide expansion is located in the intron 3—a non-coding region of TCF4—which makes the design of effective editing strategies complicated. Namely, the mutation does not result in an altered protein but rather, a toxic RNA, for which a classic genetic gene replacement is not an option; rather, either the editing of toxic RNA production or regaining the normal expression of *TGF4* gene are needed.

Applying the CRISPR-Cas9 to such mutation includes several technical challenges.

The non-coding region often expresses varying genetic structure, which represents a difficulty in finding effective guide sequences [86], resulting in inefficient binding and possible off-target cleavage. While large, repetitive DNA tracts result in difficulty in completing the excision of a large repeats, and unreliable repair mechanisms in non-dividing endothelial cells [41].

The allele-specific editing is also difficult due to the high similarity of wild-type and mutant alleles, which differ only by the repeat length [87,88].

To present a realistic picture, there is limited and indirect preclinical evidence supporting a successful CRISPR-mediated correction of TCF4 expansions in corneal endothelial cells [66,89,90].

The alternative RNA-targeting catalytically inactive or death Cas9 (dCAS9) proved a successful option in reducing toxic nuclear foci and mutant intronic RNA levels in FECD endothelial cells [91]. Other possible therapeutic strategies include the following: an antisense oligonucleotide principle could target the CUG or CAG RNA transcripts from pathological trinucleotide expansion and thus block the formation of toxic foci; the restoration of normal splicing could, as another strategy, be obtained via overexpression of the splicing factor (MBNL proteins); small molecule therapeutics could induce disruption of RNA hairpin; and some other possibilities were also suggested [41].

### 5.4. Real World Genetic Treatment for FECD

There are no clinically approved genetic treatments for FECD, nor are there any clinical trials ongoing yet.

With the latest evidence demonstrating that the pathogenic CTG trinucleotide repeat expansions in *TCF4* is a frequent genetic background in patients with the clinical picture of FECD, the CRISPR-Cas9 technology for FECD holds significant promise in two aspects [47]: on one hand, as analogous to emerging genetic treatments for trinucleotide repeat expansion disorders such as Huntington’s disease or myotonic dystrophy; and on the other hand, similar to approved genetic therapy for other eye disorders, such as retinal.

There was a patent registered proposing the cornea as a site for CRISPR–Cas9-based therapeutic approaches [92].

#### 5.4.1. DT-168

A small molecule named DT-168 was developed in the form of topical corneal therapy for FECD and aimed to inhibit the transcription of CTG18.1 into pathogenic *TCF4* RNA, which transcribes into splicing proteins initiating transcript mis-splicing (spliceopathy), which eventually results in endothelial cell death [93].

Corneal endothelial cells were cultured from donors with a clinical diagnosis of FECD who underwent endothelial keratoplasty and were exposed to the DT-168 molecule. DT-168 significantly reduced nuclear foci to levels seen in cells cultured from healthy individuals. Exposition to DT-168 molecule also improved mis-splicing in vitro in different genes. Subsequent animal experiments treating rabbit eyes twice daily for two weeks proved good tolerability and even corneal distribution; it also proved persistent corneal concentration for 24 h, with negligible plasma levels of the molecule [93].

In conclusion, DT-168 in eye drops could be useful as disease-modifying therapy attaching the most frequent genetic change in FECD.

Results of a phase I clinical study on healthy volunteers are expected in October 2025; however, there are preliminary reports of good tolerability without serious adverse events and there are no treatment discontinuations due to AEs in the trial.

A phase 2 biomarker trial of DT-168 was set to study safety, tolerability, and corneal endothelium biomarkers in FECD patients scheduled for corneal transplant surgery [94].

#### 5.4.2. Ad-Cas9-Col8a2gRNA

An animal study was performed proving efficient knock down of mutant *COL8A2* expression in endothelial cells of FECD model mice [95].

Namely, adenovirus vector was used to introduce *Cas9* gene and RNA-Ad-Cas9-Col8a2gRNA as a single intraocular injection. Using this postnatal gene editing prevented endothelial cell loss and improved their pump function without adverse effects on histological or electroretinographically exams [95].

This study proved that Ad-Cas9-Col8a2gRNA disruption of the Col8a2 start codon restores the normal endothelial cell phenotype in adult post-mitotic non-reproducing cells and could represent a medical genetic therapy of adult-onset diseases like FECD.

## 6. Discussion

Compared to the retina, the cornea, which is the outermost tissue of the eye, has the advantage of being easily accessible to different treatment delivery options such as drops [93] or intracameral injections [95]. There are also different possibilities of precise treatment evaluation via slit lamp clinical exam and advanced diagnostic methods such as specular or corneal confocal microscopy of endothelial cells, corneal optical coherence tomography, etc. The application of CRISPR/Cas9 ocular genome treatments in clinical use is on the rise, and corneal diseases will hopefully become part of this in the near future [96,97].

FECD is a devastating disease, which represents one of the most frequent reasons for the need for corneal transplantation. This procedure, however, is not readily accessible to patients in need due to global corneal tissue shortage, while procedures without donor tissue, such as DSO, are only suitable for patients with early-grade disease. Thus, a non-surgical, readily accessible, and economically affordable treatment is needed. The inherited mutations in FECD are well-researched, the cornea represents an easy to approach and immune-privileged tissue and seems very suitable for CRISPR-Cas9 therapy.

There are already molecules developed that can stop the pathological vicious circle in FECD-affected post-mitotic endothelial cells, such as the DT-168 or the RNA-Ad-Cas9-Col8a2gRNA, and have been tested in preclinical, and some as well in phase I clinical studies, and have entered phase II clinical studies [93,95]. However, there are still certain challenges as compared to already approved genetic therapies for retinal diseases [68,98]. Namely, their delivery and duration of effect, as well as clinical efficiency due to the polygenic nature of FECD are challenges to be solved.

Due to their post-mitotic state, corneal endothelial cells might be more difficult to be reached by adenoviral vectors [99], while herpes viral vector or non-viral vectors appear to be not efficient enough [100,101] to reach the endothelial cell interior.

FECD develops due to toxic gain-of-function after trinucleotide repeat resulting in protein misfolding, which is different to the retinal pigment epithelium (RPE) loss of function in the RPE65-mediated inherited retinal dystrophy [68]. FECD patients thus need long-term suppression of toxic gene activity while one-time treatment is enough to repair the pathological gene with Luxturna [68].

The polyfactorial nature of FECD will continue to be the main clinical challenge, which will eventually demand a reliable genetic test to select patients suitable for genetic treatment.

## 7. Conclusions

There are well-established treatments for the FECD but due to rising numbers of patients unmet by the tissue supply shortage and with the debilitating effect on vision and quality of life of untreated patients, the need for advanced treatment is clear.

Results of ongoing research in genetic treatment possibilities are optimistic; however, the review of the literature proved that they are still at the preclinical stage for FECD and that there are several specific challenges connected that still need to be solved before realistic clinical usage is expected.

## Figures and Tables

**Table 1:** Different genes of early and late-onset FECD.

FECD	Gene	Mutation—Significance or ACMG Classification *: 5–1	References
Early-onset	*COL8A2*	Q455K-5 L450W-5 R155Q-1 R304Q-3 R434H-3	[13,14,15,16]
Late-onset	*TCF4*	CTG repeat within third intron—pathogenic above >40–50 repeats rs613872-3 rsl 7595731-3 rs9954153-3 rs2286812-3 rs784257-3	[17,18,19,20]
	*DMPK*	CTG repeat within the 3′ UTR—not common	[21,22]
	*SLC4A11*	E399K-4 G709E-4 T754M-4 c.99–100delTC-4 E167D-3 R282P-3 Y526C-3 V575M-3 G583D-3 G742R-3 G834S-3 W240S-3 V507I-3 T434I-3	[23,24,25]
	*TCF8/ZEB1* *(chr 10p11.22)*	N78T-4 Q810P-4 Q840P-4 A905G-4 P649A-4 N696S-3 rs77516068-3 rsl49166539-3 IVS2+276-3 S234S-3 E733K-3 A818V-3 L947Stop-3	[24,26,27]
	*L0XHD1*	R547C-4 rs450997-3	[20,28]
	*AGBL1*	R1028Stop-3 c.2969G>C-3	[29]
	*KANK4* *(chr 1q25.3)*	rs79742895-3	[20]
	*LAMC1*	rs3768617-3	[20,30]
	*LINC00970/ATP1B1*	rsl022114-3	[20]
	*DNA Repair enzymes* *RAD51* *FEN1* *XRCC1* *NEIL1* *LIG3*	c.−61G>T/rs1801321)-3 c.−98G>C/rs1801320-3 rs4246215-4 c.H96A>G-4 g.46438521G>C-3rslO52536-3 rs3135967-3	[31,32,33,34]
	*Extracellular Matrix* *TGFBI* *CLU* *COL8A2*	one haplotype-3 rs 17466684-4 R304Q-3 R434H-3	[34,35,36]
	*Mitochondrial* *ND3* *TSPOAP1*	*A10398G-1* Variants-3,4	[18,37]
	*Other* *PITX2* *PTPRG* *FASLG* *KCNJ13*	g.20913G>T-3 rs7640737-3 rs 10490775 c.−671A>G-3 R162W-4	[19,36,38,39]

* ACMG American College of Medical Genetics and genomics classification for each variant (Class 5: pathogenic; Class 4: likely pathogenic; Class 3: variant of uncertain significance [VUS]; or Class 1: benign polymorphism).

**Table 2:** Chromosome loci of FECD.

FECD	Locus on Chromosome	Reference
Early-onset	1p34.3-p32 (FECD1)	[45]
Late-onset	13pter-q12.13 (FECD2) 18q21.2-q21.3 (FECD3) 20p13-p12 (FECD4) 5q33.1-q35.2 (FECD5) 10p11.2 (FECD6) 9p24.1-p22.1 (FECD7) 15q25 (FECD8)	

## Data Availability

No new data were created or analyzed in this study.

## Abbreviations

The following abbreviations are used in this manuscript:

AAV	adeno-associated viruses
Cas9	CRISPR-associated 9
CECs	corneal endothelial cells
CRISPR	clustered regularly interspaced short palindromic repeats
DMEK	Descemet membrane endothelial keratoplasty
DSO	descemetorhexis only
FECD	Fuchs endothelial corneal dystrophy
RPE	retinal pigment epithelium
